# Design of Surfaces with Persistent Antimicrobial Properties on Stainless Steel Developed Using Femtosecond Laser Texturing for Application in “High Traffic” Objects

**DOI:** 10.3390/nano13172396

**Published:** 2023-08-23

**Authors:** Albena Daskalova, Liliya Angelova

**Affiliations:** Institute of Electronics, Bulgarian Academy of Sciences, 72 Tzarigradsko Chaussee Blvd., 1784 Sofia, Bulgaria; lily1986@abv.bg

**Keywords:** ultra-short laser processing, LIPSS, nanostructuring, antimicrobial properties, surface texture

## Abstract

Metal-based high-touch surfaces used for diverse applications in everyday use, like handrails, playground grab handles, doorknobs, ATM touch pads, and desks, are the most common targets for pollution with a variety of microbes; there is thus a need to improve their antimicrobial properties, an issue which has become a challenge in recent years, particularly after the COVID-19 pandemic. According to the World Health Organization (WHO), drug-resistant pathogens are one of the main concerns to global health today, as they lead to longer hospital stays and increased medical costs. Generally, the development of antimicrobial surfaces is related to the utilization of chemical methods via deposition on surfaces in the forms of various types of coatings. However, the addition of chemical substances onto a surface can induce unwanted effects, since it causes surface chemistry changes and, in some cases, cannot provide long-lasting results. A novel approach of utilising ultra-short laser radiation for the treatment of metallic surfaces by inducing a variety of micro- and nanostructuration is elaborated upon in the current research, estimating the optimum relation between the wettability and roughness characteristics for the creation of antimicrobial properties for such high-touch surfaces. In the current study, AISI 304–304L stainless steel metal was used as a benchmark material. Surface texturing via laser ablation with femtosecond laser pulses is an effective method, since it enables the formation of a variety of surface patterns, along with the creation of bimodal roughness, in one-step processing. In this investigation, a precise approach toward developing hydrophobic stainless steel surfaces with tunable adherence using femtosecond laser-induced modification is described. The impact of basic femtosecond laser processing parameters, like the scanning velocity, laser energy, and wettability properties of the laser-processed stainless steel samples, are examined. It is identified that the topography and morphology of laser-induced surface structures can be efficiently changed by adapting the laser processing parameters to create structures, which facilitate the transfer of surface properties from extremely low to high surface wettability.

## 1. Introduction

The decreasing efficacy of antibiotics for resisting infections is becoming an increasing issue in public areas and hospitals. Future trends, predicted by the World Health Organization (WHO), foresee an increasing number (more than ten million per year by 2050) of deaths worldwide, induced by drug-resistant bacteria. In the current study, we propose a method through which to prevent microbial biofilm formation and the distribution of infections by altering the survivability of bacteria on frequently touched contact surfaces, by applying femtosecond laser-induced micro- or nanostructuring. Bacteria react very sensitively to topographical surface structures, where dimensions in the micro- and nanometre range exhibit an increased effect over bacterial cell viability [1]. Through the creation of submicron structures, a distinct decrease in contact area between the bacterial cell and the surface leads to reduced adhesion. This process could be explained as a result of the very small dimensions, less than a micro-metre, of bacterial cells. Thus, the laser-created submicron structures act as sharp protrusions, which shear apart the bacterial cell membrane and finally kill the bacteria cell. One very common metal material used in high traffic zones is stainless steel (SS). It is a widely used material in all public zones, like hospitals, hospital furniture, and subway stations; thus, there arises a necessity of applying a durable, highly resistant coating in order to achieve better antibacterial performance from SS in everyday use. SS is very widely applied for diverse interior surfaces in hospitals, and the main risk of spreading and transmitting infections is hidden in those frequently touched areas, thus creating a huge health hazard. The statistics show that hospital-acquired infections (HAIs) [2] are the most frequent cases that cause death in the USA [3]; approximately 1 out of 25 patients who have to be treated at a healthcare center acquire an infection, regardless of the health problem for which they are admitted to the hospital.

The research in the field of material design, for high and durable antibacterial properties, is still limited, and diverse types of surface finishing approaches should provide a model for the creation of a surface with persistent antimicrobial properties for the prevention of biofilm formation [4]. It is very complicated to obtain material surfaces with appropriate pathogen-free characteristics, and to simultaneously preserve specific mechanical properties. In this work, we will focus on 304 grade SS. Mostly used in public environments, it retains approx. 18% chromium (Cr), which provides corrosion-resistant characteristics. Other important points for achieving proper antimicrobial features are the following parameters: surface wettability, roughness, morphology, and chemistry [5]. Diverse studies have demonstrated that, by inducing hierarchical surface structuration via the micro/nanorange, bacterial adhesion is perturbed due to decreased surface contact area [6].

Currently, the disinfection of surfaces is performed by utilising compounds which do not have long-term stability and cannot be used as permanent protection against pathogens like Gram-negative and Gram-positive bacteria, and diverse types of viruses. In this research, an attempt will be made to study the possibilities for creating stable, durable surfaces by inducing hierarchical structures via femtosecond laser-induced ablation, which will contribute to improving the persistence of antibacterial efficacy.

A variety of methods exists for limiting the spread of different microbes. For example, Dhyani et al. designed an antimicrobial surface with long-lasting antimicrobial characteristics by incorporating plant secondary metabolites, which could be used for the rapid disinfection of pathogens, within minutes, thus influencing antimicrobial efficacy over a period of several months [7].

The connection among roughness, wettability properties, and bacterial size dimensions is essential for bacterial attachment to diverse surfaces and has to be considered for evaluation over the effectivity of a particular surface morphology against various bacterial strains. A series of studies applying laser texturing to metal surfaces has demonstrated that not only wettability, but also surface morphology affects antimicrobial properties, and the reduction in the initial rejection of microbes is an interplay between hydrophobicity and surface roughness [8].

S. Sarbada et al. [9] reported the creation of superhydrophobic surfaces on metals and polymers, utilising high speed femtosecond laser pulses. Different surface structures with micro- and nanodimensions were achieved, and their corresponding wetting characteristics, with a water contact angle (WCA) of more than 150° and a roll-off angle of less than 5°, were demonstrated on laser-processed copper and polydimethylsiloxane (PDMS) surfaces [9].

Ultra-short laser-based surface texturing possesses numerous advantages over other modification techniques. Laser-induced texturing can be performed with high precision and tunability of the laser radiation parameters, and it provides a possibility for diverse geometrical designs. For example, Cunha et al. succeeded in obtaining four main variations for surface patterning: nanostructures based on laser-induced periodic surface structures (LIPSSs); nanopillars; bimodal roughness formed from LIPSSs and overlapping micro-columns; and complex textures, which combine LIPSSs and micro-columns, with a periodic variation in column size [10].

In this research, the possibility to apply femtosecond laser treatment to obtain stainless steel surfaces with tunable wettability was investigated on AISI 304–304L/DIN1.4301-1.4307. Stainless steel is largely used for application in public zones as well as in clinical environments. The current research focuses on the investigation of the options to design highly bactericidal, durable stainless steel surfaces by employing femtosecond laser patterning. Laser processing is a single-step contactless method with the possibility of creating a large variety of surface textures [11] by precisely tuning the laser parameters [12,13,14]. Laser surface processing by employing ultra-short laser pulses can be used to design surfaces with antimicrobial characteristics by altering material surface morphological and wettability properties [15,16]. A common phenomenon associated with femtosecond (fs) laser processing is the creation of laser-induced periodic surface structures (LIPSSs) and hierarchical micro-nanostructures [17], and these nanorippled LIPSSs have been found to inhibit bacterial contamination. Anja M. Richter et al. have found a dependence of *E. coli* adhesion upon diverse spatial periods of LIPSSs. They succeeded in generating LIPSSs, varying spatial periods in the range from 214 to 613 nm on polyethylene terephthalate (PET) foils, and found a strong relation between bacterial adhesion and structure dimensions in the range of one order of magnitude [18].

In order to ensure stable antimicrobial properties, it is essential to preserve the uniformity of the surface morphology, in particular, the homogeneity and dimensions of the created micro-nanostructures, the chemical composition, and the phase preservation. Such a level of structure control usually requires several steps of surface processing, which distinctly provides properties of the substrate and forms the composition of texture designs by means of providing hierarchical roughness and uniformity. The presence of a top layer of oxidation is one of the necessary conditions for obtaining stable hydrophobicity. In this study, the employment of the femtosecond laser patterning of material surfaces allows for the combination of chemical modification and surface texturing with appropriate morphological characteristics for acquiring persistent antimicrobial properties in diverse environmental conditions. Here, we have focused on the effect of femtosecond laser patterning on SS surface durability by modifying surface roughness, wettability, and chemical stability under diverse time periods and temperature conditions. In this research, the behavior of stainless steel material was pursued by utilising several irradiation parameters and environmental conditions. The change in the surface morphology, oxidation, and temperature of the base material was examined via morphological, chemical, topographical, and wettability analysis. The estimation of the dispersion of the LIPSSs’ orientation angle will provide additional information about the achieved reproducubilty of the orientation of obtained LIPSSs. In order to completely examine the antimicrobial resistence of laser-designed surfaces against *S. aureus* and *E. coli*, long-lasting antibacterial efficacy tests against these bacteria were executed on LIPSSs’ textured surfaces and on the hierarchical textures generated at different time points and temperature conditions.

## 2. Materials and Methods

### 2.1. Femtosecond Laser Processing

The femtosecond laser texturing was performed on cold-rolled, mirror-polished bright-annealed (BA) finishing AISI 304 stainless steel (Cr—18–20%, Ni—8–10.5%) plates with dimensions of 1 × 1 cm and a thickness of 1 mm. Prior to laser treatment, samples were cleaned with 70% ethanol in an ultra-sonic bath for 10 min. After laser treatment, samples were washed with distilled water and any residual debris was removed ultra-sonically, again using 70% ethanol, prior to drying in air.

The patterned stripes were acquired with linearly polarised laser pulses delivered from a Solstice ACE (Spectra physics) laser system. The average output power of the laser system is up to 6 W, with a repetition rate of 1 KHz at a central wavelength of 800 nm and a pulse duration of 60 fs. The diameter of the focused laser beam at a Gaussian-profile laser at 1/e2 of its intensity maximum was approximately 25 μm. The surface of the metal SS sample was irradiated in dimensions of 1 cm × 1 cm. The number of applied laser pulses (*N*) accumulated on one irradiated spot is related to the scanning speed (*V*) Equation (1):

(1) $$N=\frac{\pi .D.\nu }{4.V\alpha }$$

where $\alpha =\frac{∆}{D}$.

The overlap ratio is defined using the formula below (Equation (2)):

(2) $$\eta =1-\frac{V}{\left(\nu .D\right)}$$

where *D* is the diameter of the laser spot, *V* is the scanning velocity, and *ν* is the pulse frequency.

**Figure 1:**
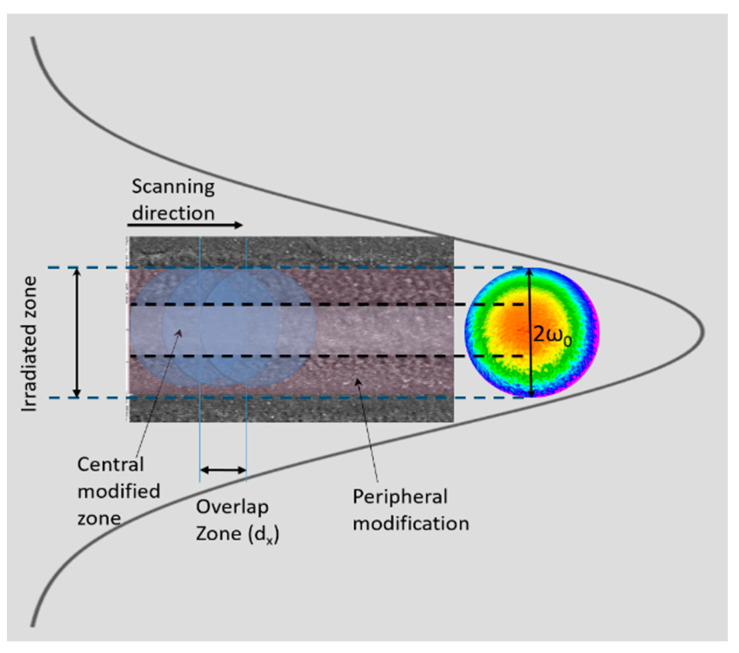
Schematic representation of the two different areas that formed the morphology of the created structures. The modification zone is composed of two separate areas: the central one and the peripheral one. The formation of the two different zones can be explained via the diverse fluencies along the Gaussian beam distribution.

The emitted laser radiation was directed towards a scanning XY translation stage for performing sample surface patterning (Figure 1).

The overall scanning design consisted of the creation of parallel lines on the sample surface, separated by an interval of 50 μm. Assigning V to the scanning velocity, several experimental conditions were applied (Table 1).

After laser processing, the treated surfaces were sonicated for 20 min in 70% ethanol at room temperature, and subsequently washed with dH20 and dried in air for further surface analysis.

### 2.2. Surface Characterization: Morphological, Wettability, and Elemental Analysis

To evaluate the morphology of the produced surface patterns, scanning electron microscopy (SEM) analysis was performed using scanning electron emission microscopy—SEM (Hitachi SU5000 + EDS/WDS Thermo Scientific ANAMAT or LYRA I XMU). Images were acquired at three different magnifications: 1000×, 5000×, and 10,000×, and the accelerating voltage (HV) was kept to 20 kV; no sputtering was applied due to the metal nature of the examined specimen. The energy-dispersive spectroscopy (EDS) analysis was obtained at a magnification of 11,000×. To analyse the obtained LIPSSs, the SEM images were additionally explored using discrete two-dimensional fast Fourier transform analysis (2D-FFT). The two-dimensional (2D) fast Fourier transformations (FFT) were achieved via open-source software (Gwyddion, Version 2.55).

The estimation of DLOA was performed from SEM images in the ImageJ programme. The acquisition of dispersion in the LIPSS orientation angle (DLOA) was completed in the Orientation Distribution module of the Orientation J plug-in by applying the Riesz filters tensor with a local window of 1 pixel.

To study the change in the wettability properties of the laser-processed surfaces, the characterization of static contact angles (CA) was performed using the contact angle metre (KRUSS DSA100) under room conditions. For water contact angle measurement, the static contact angles were evaluated using the sessile drop method which invoved a drop volume of 2 μL using distilled deionized water and a stainless steel 0.5 mm needle. The WCAs were measured at room temperature conditions t = 20 °C. The measurements of CA consisted of the evaluation of each CA every econd for the first minute of the measurement and every 60 s for the next 2 min until it reached an equilibrium state, for a total period of 180 s. The SS samples were analysed before and after laser structuring, and the experiments were performed in the air with acquisition via the video-based optical contact angle measurement device DSA25 Drop Shape Analyzer (KRÜSS GmbH, Hamburg, Germany). Contact angles were calculated using ADVANCE software V.1.7.2.2 (KRÜSS GmbH) by fitting the drop profiles to the Young–Laplace equation. The dynamic WCA was obtained as an average value of 5 separate measurements.

The estimation of the surface roughness profile was performed via a 3D optical profiler, Zeta-20 (Zeta Instruments, KLA, Milpitas, CA, USA), at 20× magnification. ProfilmOnline software (https://www.profilmonline.com, accessed on 23 of March 2023) was used for the improved visualisation of the 3D colour images.

The ageing effects on the laser-processed samples were explored by verifying the change in the X-ray diffraction analysis (XRD) spectra of the same sequence used in the above-described measurements for 4 different time points: after 1, and 30 days in air. In order to maintain reproducible conditions of surface elemental composition, no cleaning procedure was applied to clear possible pollutants from environmental exposure.

The effects of laser processing on the phase of the material were analysed via micro-Raman and X-ray diffraction studies. The Raman spectra were acquired via a micro-Raman spectrometer (LabRAM HR Visible, Horiba, Kyoto, Japan) equipped with a He–Ne laser (633 nm) and a microscope (BX41, Olympus, Tokyo, Japan).

For the identification of possible phase transformations of SS after laser processing, an XRD analysis was performed within the range of 5–80°θ2 (step size of 0.05°θ2, in a continuous scan mode, and with a counting time of 4 s) utilising a Philips PW1050 X-ray diffractometer system (Philips, Amsterdam, The Netherlands), possessing a secondary monochromator for the diffraction beam and a copper anode. The phase identification was obtained via QualX2 software, through the Crystallography Open Database.

### 2.3. Antimicrobial Studies

The antimicrobial tests were performed with two types of bacteria strains: Gram-positive and Gram-negative bacteria. On stainless steel tiles that were pre-cleaned with alcohol and irradiated under UV for 4 h, *E. coli* or *S. aureus* inoculums were deposited and were instilled at a concentration of 1 × 10^6^. The inoculums and cultivation of biofilms were conducted in tryptic soy *broth* TSB medium. The biofilms were cultured on a plate under static conditions for 24 h. For the observation of morphology via SEM, the following procedure for bacterial cell fixation was used: washing with 0.1 M sodium cacodylate, fixation with 4% glutaraldehyde for 1 h, washing with cacodylate buffer, post-fixation of the samples in 0.1% osmium tetroxide, and dehydration in an ascending ethanol series. Finally, the samples were dipped into carbon-conductive tape, and afterwards they were sputter-coated with gold.

## 3. Results

### 3.1. Evolution of Surface Morphology

**Figure 2:**
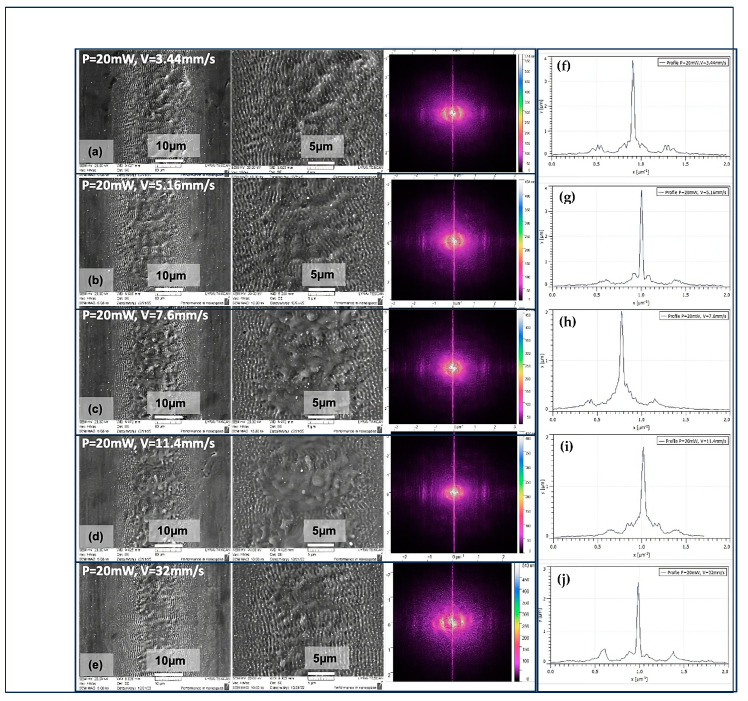
SEM images (scale bars 10 and 5 μm) and images of the corresponding 2D FTT transformation of the produced patterns on SS surfaces in relation to the laser power and scanning velocity: (**a**–**e**) P = 20 mW, V = 3.44, 5.16, 7.6, 11.4, and 32 mm/s, (**f**–**j**) cross-sectional images of LIPPSs’ orientation angle distribution under a variation of V = 3.44, 5.16, 7.6, 11.4, and 32 mm/s at constant P = 20 mW.

**Figure 3:**
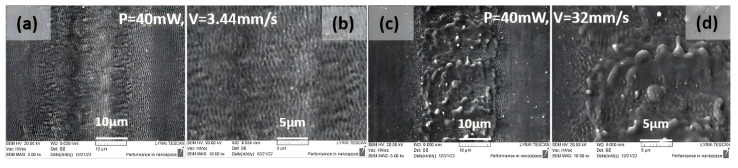
SEM images of the produced patterns on stainless steel surfaces in relation to the change in the scanning velocity: (**a**,**b**) P = 40 mW, V = 3.44, (**c**,**d**) P = 40 mW, V = 32 mm/s.

SEM micrographs of ultra-short laser-processed 304 L stainless steel samples reveal that the changes in surface morphologies strongly depend on the interplay between several laser parameters: the scanning velocity, laser power, and distance between separate rows. In Figure 2 and Figure 3, the SEM images of stainless steel surfaces obtained under irradiation with diverse values of scanning velocity (V) for two laser powers are represented. The evolution profiles of the obtained laser structures clearly demonstrate the formation of laser-induced periodic surface structures (LIPSSs) independently of the applied laser power and in the range of scanning velocities V = 3.44, 5.16, 7.6, 11.4, and 32 mm/s of groups G1 and G2 (Table 1). The nano-ripple orientation was detected to be parallel in accordance with the direction of laser beam polarisation. Their typical length is approximately 780 nm. In the SEM images shown in Figure 2, it can be seen that by increasing V from 7.6 mm/s to 32 mm/s, the central part of the structured area starts to exhibit signs of melting (Figure 2c–e).

Such a type of formation is also visible for higher laser power P = 40 mW (Figure 3).

The evaluation of LIPSSs’ properties was performed via 2D FFT transformation from the SEM images. For the velocities from 3.44–32 mm/s, the cross-sectional images in the Fourier plane (Figure 2f–j) reveal the indications of two symmetrical peaks on the sides of the central one. These mirror-like peaks can be attributed to low-spatial-frequency LIPSSs (LSFLs). This observation suggests that the origin of LIPSSs has one period. From the acquired 2D FFT images in Figure 2a–e, the produced LIPSSs have one orientation direction, and it is possible to bridge the central peak with the two peaks within a single line propagating through the central peak. From the 2D FFT map, the LIPSSs’ periodicities were obtained by estimating the distance between two symmetric peaks, fitted by a Gaussian function. The average periodicity of LIPSSs was approx. ɅLSFL = 770 nm. Variable surface structures have been obtained by changing the scanning velocity towards lower V from 1 mm/s to 0.1 mm/s (Section 3.1).

**Figure 4:**
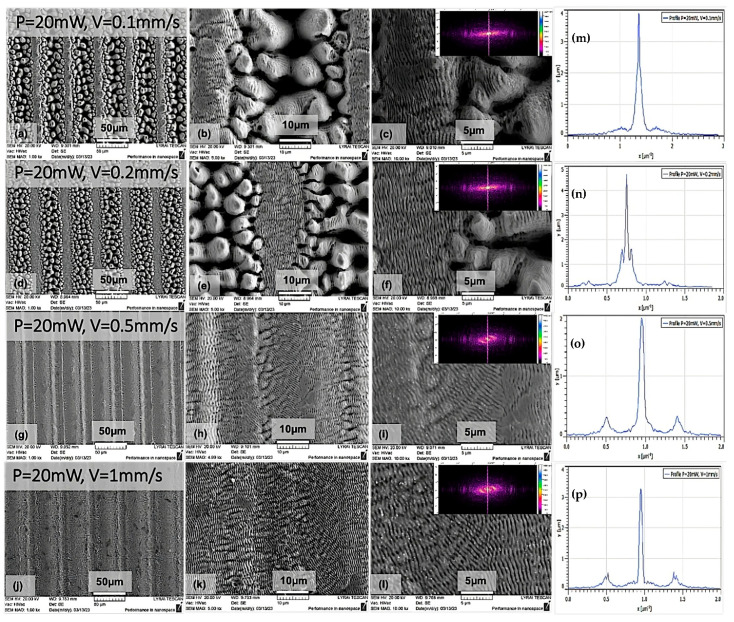
SEM micrographs of the SS surfaces: variation of the LIPSS formation on stainless steel as a function of the scanning velocity (V). Each SEM image contains a 2D FFT inset and corresponding profiles (**a**–**l**); cross-sectional graphs of LIPSSs’ orientation angle distribution under variation of V from 0.1mm/s to 1mm/s, at constant P = 20mW (**m**–**p**).

From Figure 4, it can be clearly distinguished that, when processing is performed with lower values of scanning velocities, the formation of pyramidal, elongated, pillar-like patterns evolves, which are superimposed via a quantity of laser-induced periodic nanostructures (LIPSSs). Some of the nanoscale structures are oriented in diverse directions (Figure 4h,i,k,l). The formation of dual roughness is observed due to the combination of micro-pillars and LIPSSs, which surmount the micro-columns and which are also present in the side zone surrounding the pillar pattern. Further observation of surface changes exhibited a smooth transition of the column-like structures to periodic nanostructures with more shallow depths by increasing the scanning velocities to 0.5 and 1 mm/s (Figure 4g–l). The overlay of both types of morphologies is formed due to the Gaussian intensity distribution in the cross-section of the laser beam (Figure 4i,l). With the decrease in the scanning speed to 0.2 and 0.1 mm/s, two zones can be distinguished: the area with micro-pillars that represents the scanning path, followed by a zone covered mainly with LIPSS formations (Figure 4a,d). It is observed that the generation of LIPSSs at the centre of the irradiation zone was oriented parallel to the laser polarisation direction of LIPSS || (Figure 4a–f). With the increase in the scanning velocity, the polarization direction of LIPSSs tends to change its orientation (Figure 4g–l). Overall, the simultaneous formation of LIPSSs with diverse orientations to the parallel (||) laser polarisation direction was monitored; this was mostly visible at the central part of the irradiated trace, and LIPSSs shifted to an angle with respect to the laser polarisation orientation.

**Figure 5:**
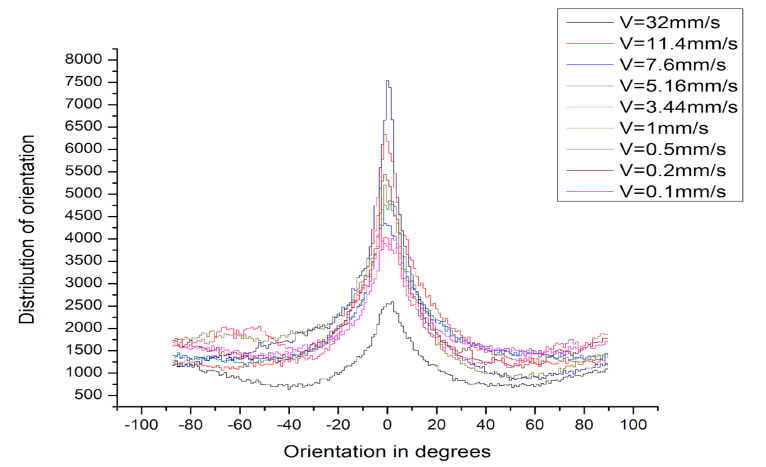
DLOA (δθ) distribution for P = 20 mW of LIPSSs’ covered zones on stainless steel under variation of different velocities ranging from V = 32 1 mm/s.

Moreover, from the performed 2D FTT analysis, the formation of high-spatial-frequency LIPSSs (HSFLs) can clearly be distinguished for 0.1 mm/s velocities, which gradually changes to LSFLs from V > 0.2 mm/s. The spatial period of HSFLs was approx. Ʌ < λ/2 (396 nm). From V > 0.2 mm/s, the generation of LSFLs dominates Figure 3d–l. The regularity of LIPSSs can be defined via the dispersion of the LIPSSs’ orientation angle (DLOA). For the obtained LIPSSs, the average DLOA value is in the range of: δθ~9° ± 4°; this corresponds to the creation of nanostructures on stainless steel with high regularity and is in agreement with findings of other groups who have obtained similar DLOA values for SS [19]. For the determination of the DLOA, a tensor analysis of the obtained SEM image was used. From the captured SEM micrograph, the angular orientation of each pixel on the image was defined, and subsequently, the pixel’s angular distribution was acquired, which corresponds to the value of (DLOA) δθ. The half-width at a half-maximum was calculated from the plotted angular distributions δθ (Figure 5).

This parameter characterises the straightness of the LIPSSs. The DLOA parameter was evaluated trough the Orientation J plug-in created for the open-source software ImageJ (1.52 v). For the evaluation of the DLOA from the SEM images, the magnification was kept at 10,000×.

### 3.2. Roughness Analysis

One of the main approaches to influencing the wetability characteristics is to change the roughness properties of the material. The arithmetic average height (R_a_) is the main roughness parameter, which can be defined by the mathematical description in Equation (3):

(3) $$Ra_{\;}=\;\frac{1}{l}\cdot \int_{0}^{l}\left|y(x)\right|dx$$

where *l* is the length of the profile.

This parameter represents the absolute changes in the surface roughness irregularities around the mean line with a specific length of sampling. It is easy to define and measure, and provides information about surface height deviations.

**Figure 6:**
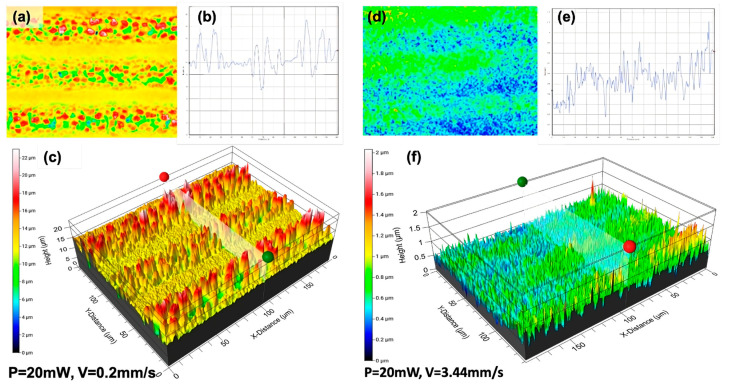
Optical profilometer 2D, cross-section, and 3D images of formed micro and nanostructures (**a**–**c**) P = 20 mW, V = 0.2 mm/s; (**d**–**f**) P = 20 mW, V = 3.44 mm/s. The red and green balls indicate the beginning and end (respectively) of the section where R_a_ was measured. Several sections selected using this method were used to average the R_a_ value.

To study the relationship between surface morphologies and applied laser parameters, the processed surfaces were examined using a 3D optical profilometer to acquire 3D profile images of surface patterns. A series of optical profilometer images showing the evolution of surface morphology with the increase in v when P = 20 mW, V = 0.2, and 3.4 mm/s are shown in Figure 6.

It can be seen that the surface roughness increases with decreasing V. The structure formation shown in Figure 6c has higher roughness values in relation to other sample surfaces processed with increasing scanning speed (Figure 6f). The obtained surface roughness has an approximate constant deviation of about 0.18 µm when the speed is increased by more than 5 mm/s.

**Figure 7:**
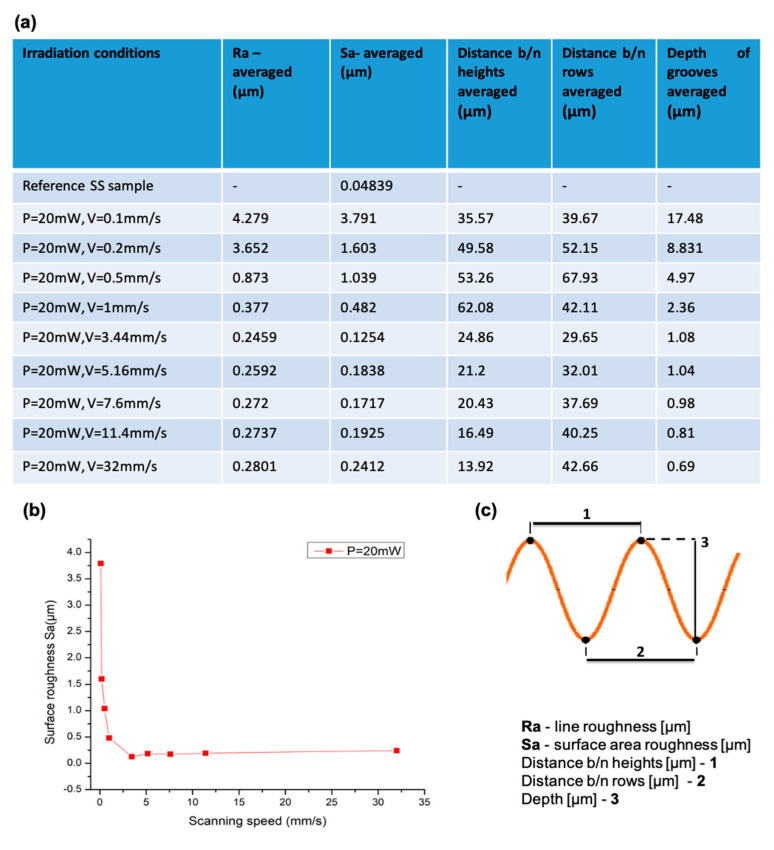
Surface roughness deviation of a laser-textured SS surface at (**a**) a list of corresponding measured surface profiles (Ra, Sa, averaged distance between two separate rows, averaged distance between two neighbouring grooves); (**b**) different scanning velocities ranging from 0.1 mm/s to 32 mm/s at laser power P = 20 mW; and (**c**) a schematic representation of the different measuring modes.

**Figure 12:**
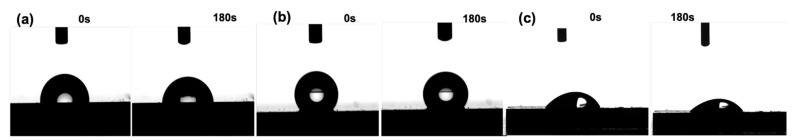
Snapshot images of WCA behaviour as a function of time for laser-processed samples: (**a**) control SS sample at t = 0 s, WCA = 97.2°; t = 180 s, WCA = 74.3°; (**b**) t = 0 s, WCA = 132.4°; t = 180 s, WCA = 128.9°, P = 20 mW, V = 3.44 mm/s; and (**c**) t = 0 s, WCA = 44.7°, t = 180 s; WCA = 39.8°, P = 20 mW, V = 0.2 mm/s.

The gradual decrease in surface roughness (Figure 7b) has slight deviations in the order of 0.05 µm, and it retains its stability. This observation of roughness reduction could be related to the change in surface morphologies (obtained from the SEM images), which become more plain with the increasing V. The surface roughness grows from 0.2 µm to 3.79 µm, and at the same time, the WCA decrease is monitored for the hybrid structures from 97.2° to 44.7°. These results differ from findings from other groups who observed a link between roughness increases and the increases of WCA [20]. In our case of hybrid structures, the water partly enters into the structures gaps, as demonstrated in Figure 12c. A general tendency of a gradual roughness decrease with an increase in scanning speed is observed (Table 2).

### 3.3. Chemical Analysis

**Figure 8:**
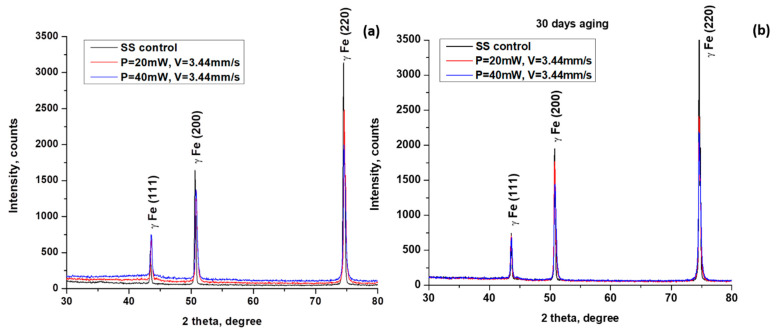
XRD patterns of laser-processed samples at two laser powers: (**a**) P = 20 and 40 mW, and V = 3.44 mm/s samples and control; (**b**) after 30 days of ageing.

Ageing effects were determined on laser-irradiated samples via XRD spectra on the 1st and 30th days in air. To avoid surface contamination and a change in composition, no cleaning was performed before the analysis. The durability properties of the developed morphological structures were put to the test over an extended period of time. As a result, after 30 days of observation, no morphological modifications were found in the produced structures. Figure 8 below displays the X-ray diffraction (XRD) results of laser-processed stainless steel under two different conditions: in situ analysis immediately during the first hours of maturation and after 30 days of ageing.

The XRD spectra for laser-processed stainless steel samples for both conditions exhibit typical phases for the three most representative peaks appearing at 2θ = 44°, 51°, and 75° (Figure 8a,b), which are also detected by other groups [21,22]. The XRD patterns are equal for both types of conditions for each parameter selection and also after 30 days of ageing. Only a small deviation in peak intensities is visible with respect to the control sample. The detected peak at 2θ = 44° represents the appearance of the magnetite phase. Those findings are in accordance with the results reported by Piotr Dywel et al. [23]. It is important to note that no new phases can be detected after laser processing.

**Figure 9:**
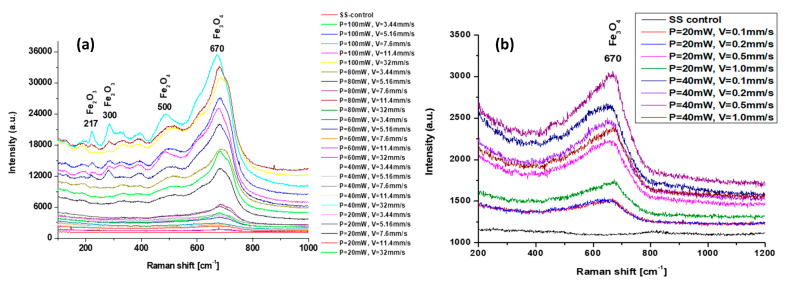
Micro-Raman spectroscopy analysis of stainless steel areas of femtosecond laser-treated areas with different scanning velocities and applied laser powers (**a**) V = 3.44–32 mm/s, P = 20, 40, 60, 80, and 100 mW, in relation to the control (non-laser-processed sample); (**b**) V = 0.1 mm/s, 0.2 mm/s, 0.5 mm/s, and 1 mm/s at P = 20, 40.

The phase composition was also examined using micro-Raman analysis. The Raman spectra contain some deviations for irradiation conditions with high and low scanning velocities (Figure 9a,b). The peaks appearing at 217 cm^−1^, 300 cm^−1^, and 400 cm^−1^ could be assigned to the formation of the α–Fe_2_O_3_ phase for P > 60 mW; those peaks are not present at Raman spectra with lower laser power and at low scanning velocities for the chosen group of applied laser power P = 20 and 40 mW (Figure 9b). For all spectra, the characteristic peak at 670 cm^−1^ was detectable, which corresponds to the Fe_3_O_4_ formation of magnetite.

### 3.4. Wettability Analysis

**Figure 10:**
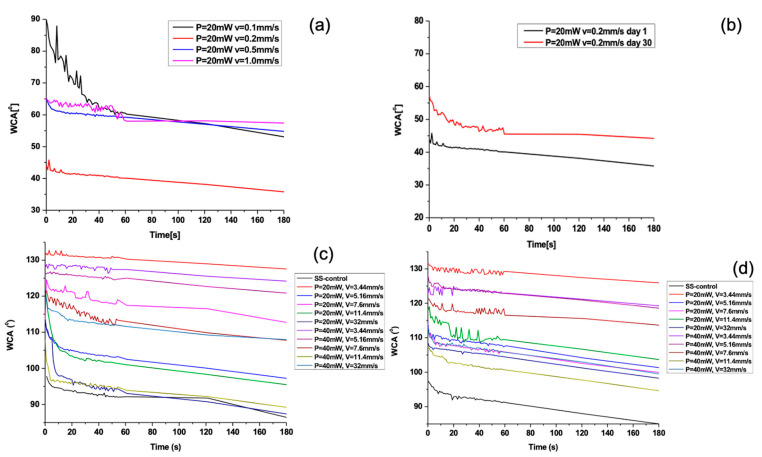
Water contact angle dependence with respect to diverse scanning velocities for SS material: (**a**) WCA for V = 0.1, 0.2, 0.5, and 1 mm/s at P = 20 mW; (**b**) V = 0.2 mm/s, P = 20 mW, on the 1st and 30th days after laser texturing; (**c**) WCA for V = 3.44, 5.16, 7.6, 11.4, and 32 mm/s for P = 20 and 40 mW; and (**d**) WCA for V = 3.44, 5.16, 7.6, 11.4, and 32 mm/s for P = 20 and 40 mW after 30 days of ageing.

The achieved laser-induced morphological changes of the 304 L stainless steel surface and the proportion of oxygen content influenced the wettability properties. The surface wettability could be modified by changing the surface roughness and chemistry. The surface wettability of stainless steel samples was analysed by measuring the WCA before and after laser processing. In Figure 10 below, the static WCA graphs, taken at the 1st day of laser exposure (Figure 10a,c) and at the 30th day (Figure 10b,d) are presented as a function of time for several scanning velocities.

**Figure 11:**
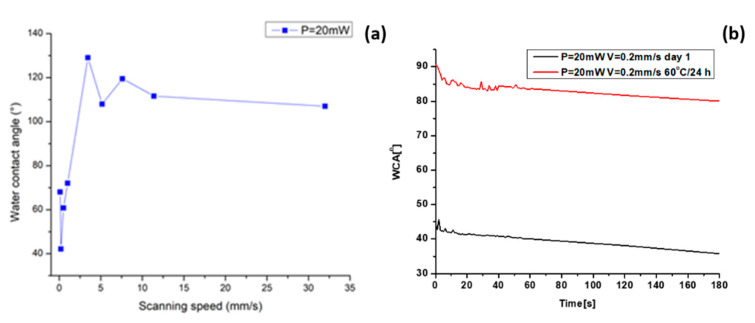
Relation between water contact angles (WCA) of a water droplet on the laser-processed SS surfaces at different scanning velocities (**a**). Selected representative graph of the dependence of WCA at two different conditions—WCA on the 1st day after the laser processing of stainless steel and WCA after heating at 60 °C for the time period of 24 h (**b**).

The increase in scanning velocities leads to a general trend of a slight increase in CA. The AISI 304—304 L initially exhibits a hydrophobic state, which becomes more hydrophobic after the laser processing with increasing scanning velocities (Figure 11a).

After femtosecond laser texturing with lower scanning speeds (<1 mm/s), the surface changes its wettability properties to hydrophilic (Figure 12c).

**Figure 13:**
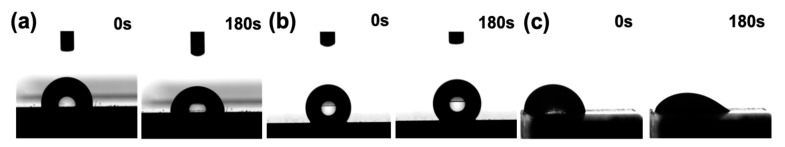
Snapshot images of water CA behaviour after 30 days of ageing as a function of time for laser-processed samples: (**a**) control SS sample at t = 0 s, WCA = 96.4°; t = 180 s, WCA = 73.7°; (**b**) t = 0 s, WCA = 131.8°; t = 180 s, WCA = 128.7°, P = 20 mW, V = 3.44 mm/s; and (**c**) t = 0 s, WCA = 54.2°, t = 180 s; WCA = 45.1°, P = 20 mW, V = 0.2 mm/s.

In Figure 13 below, the static water CA values acquired 30 days after laser processing at two different time points are shown.

**Figure 14:**
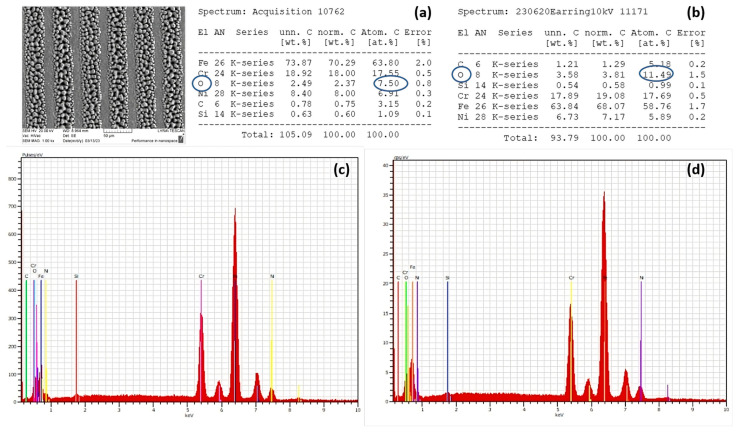
SEM/EDS analysis and corresponding values of wt.% of different elements of laser-processed sample SS sample (P = 20 mW, V = 0.2 mm/s) after treatment at a high temperature (60 °C) for 24 h: (**a**,**b**) wt.% percentages of elemental composition before and after heat treatment; (**c**,**d**) representative EDS spectra of SS from elemental distribution before and after temperature exposure. A blue circle is assigned for the comparisson of oxygen content in EDS analysis before (**a**) and after laser treatment (**b**).

**Figure 15:**
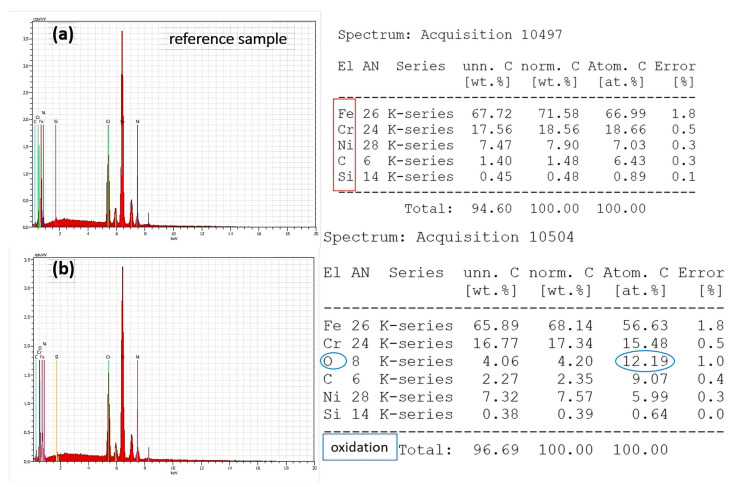
SEM/EDS analysis and corresponding values of wt.% of different elements of a laser-processed stainless steel sample: (**a**) reference non-treated SS sample; (**b**) laser-processed sample at P = 40 mW, V = 5.16 mm/s. A blue circle is assigned to detect the oxygen content in EDS after laser treatment in relation to the reference sample.

The results showed slight deviations in relation to day 1 in the WCA values, 30 days after laser patterning. Moreover, for lower scanning velocities (Figure 13c), an increase in the WCA values towards more hydrophobic behaviour is monitored in reference to WCA measurements on the first day (Figure 12c). The obtained measurement of the WCA after 30 days showed that the effect of ageing is expressed in increasing the values of the WCA. Under the examined conditions, the static WCA exhibits a strong dependency on the specific micro-structure and possesses a tunability with respect to the achieved surface structures. The obtained LIPSSs exhibit a water-repellent effect, as demonstrated by obtaining large values of the CA of water drops. As the scanning velocity further decreases, the observation of the appearance of pillar-like structures becomes clearer, and the interaction between the water droplet and the surface suddenly changes due to the rapid decrease in WCA values, turning the surface into a hydrophilic one. However, a change in wettability state was monitored, as shown in Figure 11b, after heating the sample for 24 h at 60 °C. This suggests that the temperature influence affects the 304 L stainless steel surface’s wettability, which is one of the specific requirements when designing a durable antimicrobial surface. This observation could be related to the basic observation from the EDS spectra, where an increase in oxygen content after heating (Figure 14d) is detected in comparison to non-heated samples (Figure 14c). Furthermore, similarly, after an increase in applied laser power from P = 40 mW, increasing the O content is also monitored (Figure 15b).

### 3.5. Bacteria Adhesion on Laser-Structured Stainless Steel

**Figure 16:**
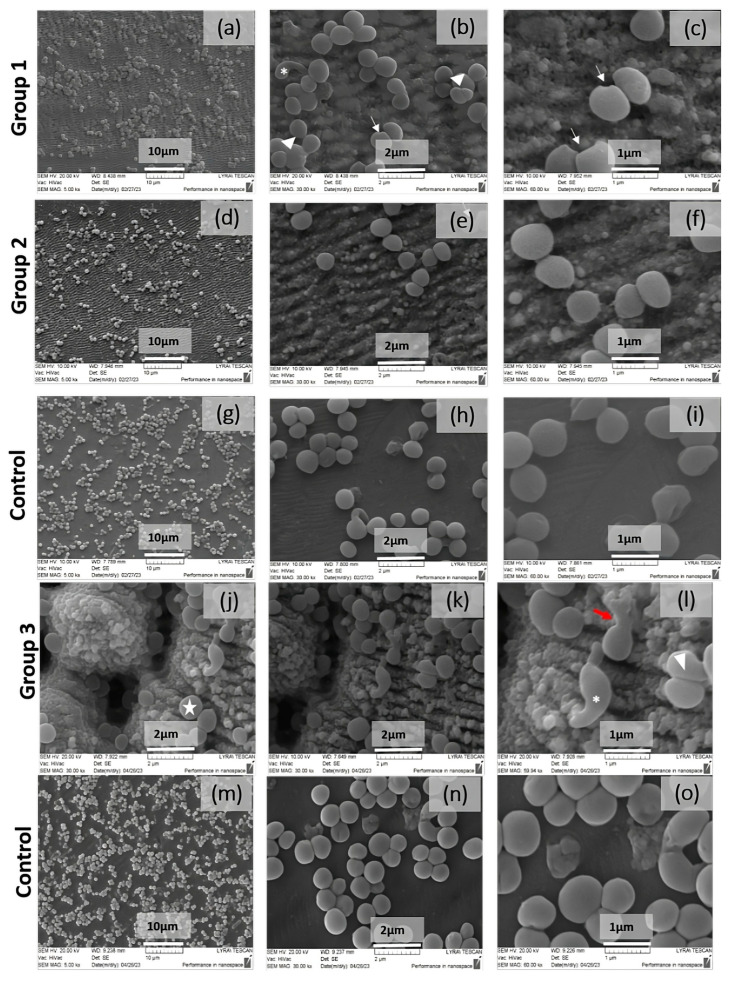
SEM images of strain *S. aureus* 29213 obtained after 24 h of incubation on laser-processed stainless steel samples from four groups: Group 1: (**a**–**c**) laser-patterned: P = 40 mW, V = 3.44 mm/s. Group 2: (**d**–**f**) laser-patterned P = 20 mW, V = 3.44 mm/s, (**d**–**f**) reference samples. Group 3: (**g**–**l**) P = 20 mW, V = 0.2 mm/s, (**m**–**o**) reference samples. (*) elongation of one cell pole type “star-like”; “large star”: grouping of the bacterial cells; “triangle”: formation of the daughter cells; cell type “arrows-like”: presence of unipolar intussusceptions.

On the basis of the presented morphological results from the cultivation of *S. aureus* on laser-structured samples, a comparative analysis can be made for the exfoliating (peeling) effect of the substrate in the case of stainless steel on the biofilm formation in the case of the strain *S. aureus* 29213. In the control sample (Figure 16g–i,m–o), we observed cells and cell clusters, typical for the formation of biofilm consortia, located on the entire surface of the material. In contrast, the laser-treated samples showed a significant reduction in the number of cells (Figure 16a–c,j–l) compared to the control and the presence of a single rearrangement of the cell monolayers.

Bacterial attachment tests demonstrate that the produced nanorippled surfaces drastically influence the adhesion of *E. coli* and depend on the design and dimensions of the LIPSSs.

**Figure 17:**
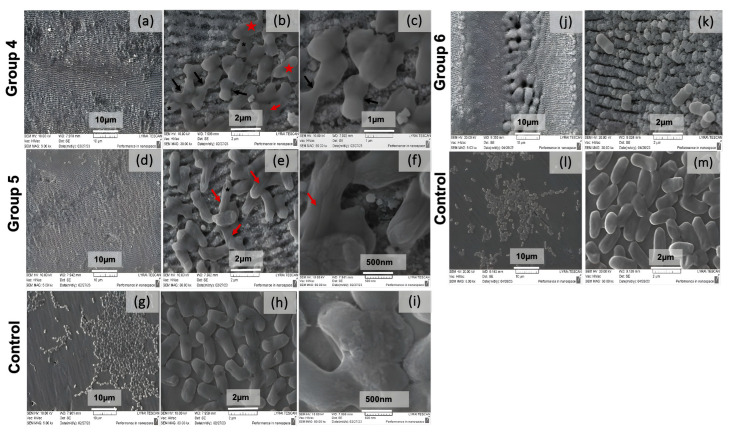
SEM images of *E. coli* adhesion after 24 h of incubation on processed SS surfaces in the form of LIPSSs (**a**–**f**) and in the form of micro-pillars with the formation of LIPSSs (**g**,**k**) in relation to the reference non-laser-processed area (**g**–**i**,**l**,**m**). Several groups were created: Group 4 (**a**–**c**): P = 40 mW, V = 3.44 mm/s. Group 5 (**d**–**f**): P = 20 mW, V = 3.44 mm/s. Group 6 (**j**–**k**): P = 20 mW, V = 0.2 mm/s. “large star”: grouping of the bacterial cells; “black arrows”: process of cell division without a clearly defined cell septa; “red arrows “: cells with intussusception along the entire length; “black star”: cells with unipolar indentations; and “red star”: synthesis of exopolysaccharide substances.

The results of the bacteria adhesion tests performed for 24 h, shown in Figure 17, exhibit a considerable decrease in the quantity of *E. coli* bacteria cells detectable on two chosen laser-textured stainless steel surfaces. Both types of prepared surfaces demonstrate antibacterial efficiency against the tested *E. coli* strain; however, the structures obtained with a lower scanning velocity regime reduced bacteria spreading and bacteria growth drastically (Figure 17j,k).

The enhancement of the antibacterial properties of SS surfaces via ultra-short laser-induced texturing could be a result of several reasons: on the one hand, the surface oxidation, and on the other, the change in surface roughness and wettability.

## 4. Discussion

The femtosecond laser treatment of 304 L stainless steel at high scanning velocities induces the formation of laser-induced periodic surface structures (LIPSSs) on a nanometric scale. The wettability measurement performed on this type of surface covered with nanometric structures has a WCA θ < 140°. By further decreasing the scanning velocities, LIPSSs and periodic pillar-like spikes with micron dimensions start to evolve, surmounted by LIPSSs. The occurrence of micro-pillar morphology could be attributed to the formation of hydrodynamic processes in metals in the course of extreme heat deposition [24]. The establishment of a thin layer of melt and the organisation of the material due to surface tension and its fast solidification lead to the generation of micro-columnar structures. Among a number of explanations for the LIPSSs’ formation mechanism, the studies of P. Terekhin et al. have explored the LIPSSs’ formation mechanism after single-pulse laser irradiation via the hybrid atomic-continuum model (MD-TTM), where the MD approach is applied to describe the appearance of transient states of matter due to the laser-induced structural dynamics. They have identified two components of LIPSS formation: surface plasmon polariton (SPP) excitation, which causes the deposited energy modulation, and material reorganisation, which provides the final LIPSS morphology [25].

The antimicrobial properties of LIPSSs, which were demonstrated in these studies, could be described via the repulsive forces that increase at the nanotextured areas. As the surface irradiation is performed in the regime of a further increase in laser scanning speed, the repetition of pulses per irradiated spot causes melting in the central part of the modified area, and the regularity of the periodic structure diminishes. In the studies of P. Lickschat et al. [26], the removal efficiency and the ablation quality of stainless steel have been found to be strongly dependent on the fluence and pulse duration. The smoothing effect was monitored and explained via the formation of a melting film. Moreover, the melting depth caused by the burst mode contributes greatly to the smoothing. Zhang et al. [27] have determined that three key parameters must be maintained in order to effectively construct reproducible LIPSSs: improving the periodic deposition of laser energy, reducing residual heat, and avoiding debris deposited on the ablation zone. With temporally tailored ultra-fast pulses, this is possible. The LIPSS uniformity could be changed by adjusting the subpulse interval, pulse count, and subpulse energy distribution [27].

Another study by Zhang D. et al. demonstrated that liquid flow, including liquid vortexes produced by shockwaves or due to the collapse of cavitation bubbles during fs laser ablation, plays an important role in causing the movement of molten layers [28].

In most cases, the transformation of surface topography caused by laser processing is accompanied by a change in surface chemistry. For this reason, the XRD chemical characterization, EDS, and micro-Raman characterization of the selected LIPSSs and hierarchical structures on 304 L stainless steel were carried out. From the performed analysis of micro-Raman spectra, under the variation of laser power from P = 20 mW to P = 100 mW, the detection of peaks of ferric oxide/hematite at 217 and 300 cm^−1^, and at 500 cm^−1^ (Fe_2_O_4_), which can be assigned to rust formation, occurs for laser powers more than P > 40 mW. These observations are in complete agreement with the findings from the EDS analysis, where an increasing percentage of oxygen content (Figure 15b) was detected with an increment of applied laser power of more than P > 40 mW. The obtained XRD results on the 1st day after laser processing and after the 30th day after laser treatment showed the slight amorphization of the treated surface due to the registration of the amorphous halo under the peak with Fe (111) (Figure 8a), which with time is not detectable (Figure 8b).

Upon closer analysis of antimicrobial research, *S. aureus* bacterial cell morphology, at higher magnification (Figure 16b,c,e,f,k,l), including the presence of both typical and aberrant cell morphology, was noted. In the treated samples of group 1, cells with division disorders and altered morphology were observed. Morphological defects were reported in the process of division and the formation of the daughter cells—triangles. Cells with an irregular shape and elongation of one “star-like” cell pole type were also found. There were also cells with an atypical coccoid shape and disturbances in the integrity of the outer surface, expressed in the presence of unipolar intussusceptions of the”arrows-like” type were visible. In the samples of group 2, apart from a reduction in the biofilm biomass and single invaginations in the bacterial surface, more significant morphological damages were not observed.

The SEM analysis of the formed biofilm community of *S. aureus* on the control and laser-processed samples within group 3 showed the presence of biofilm monolayers and/or cell clusters of cells with a characteristic ubiquitous distribution on the substrate. Comparatively, in the treated sample, areas of non-adherent cells on the substrate and a loose distribution were reported, which is an indication of the presence of an exfoliating effect as a result of the treatment (Figure 16k,l). The analysis of the morphology of S. aureus bacterial cells is similar to the previous results from treatment within groups 1 and 2. A conclusion can be made here about the recognition of morphological aberrative defects in the division process and the appearance of bacterial cells with an irregular shape and elongation of one “star-like” cell pole type. The typical grouping of the bacterial cells in a chain—a large star—was also found. Analogous to the previous treatment (groups 1 and 2), morphological defects were also found here in the process of the division and formation of the daughter cells—a triangle. In contrast, in the new treatment parameters, we report the single presence of a bacterial finding with the leakage of cell contents as a result of a possible rupture of the cell membrane (Figure 16k,l).

From the morphological analysis performed on the biofilm formation of strain *E. coli* 25922, a monolayer cell distribution is clearly visible in the control sample (Figure 17g–i,l,m). In contrast, on the laser-processed substrate, with a high probability, about 70–80% exfoliating activity on the bacterial consortia is reported. The biofilm formed on the treated sample is represented by isolated biofilm islands of clustered cells, between which there are empty areas with the presence of single, possibly deformed cells (Figure 17). The analysis of the morphology of the bacterial cells of *E. coli* 25922 showed that in group 4, as a result of the laser treatment, various morphological changes in the cell ultra-structure are reported, including damaged, deformed, and underdeveloped cells with defects in the process of division without a clearly defined cell septa—black arrows. Other morphological abnormalities, more distinctly expressed during treatment in group 5, are the presence of cells with intussusception along the entire length—red arrows. Probably as a result of the treatment, unipolar indentations are found on the cell surface in both groups—a black star. In group 4, the synthesis of exopolysaccharide substances is noticed (Figure 17d–f)—red star.

Based on the comparative analysis of the biofilm community of *E. coli* 25922 from the two treatments (high and low scanning velocities), it can be concluded that a significant exfoliation effect in the treated steel was found compared to the control sample. The finding of biofilm clumps and island formations present in the control sample compared to the treated sample showed a significant effect of the parameters used. The reduction of cells is decreased to the presence of single cells or groups of two to three on the treated substrate (Figure 17j,k).

## 5. Conclusions

This study aimed to design micro- and nanoscale hierarchical surface structures on 304 L stainless steel substrates, processed with femtosecond laser pulses, for the creation of durable antimicrobial properties for diverse applications in public zones with high traffic. The effect of femtosecond laser treatment on the formation of stable morphological structures on 304 L stainless steel was investigated. Due to the application of diverse scanning velocities, two types of micro-structures were designed: LIPSSs and pillar-like columns surmounted with LIPSSs. The surface wettability changes with the variation of surface roughness, which leads to an alteration of the bacterial/surface interaction mechanism, which was also monitored. The long-term wetting characteristics of 14 selected scanning velocities and two laser powers were investigated and showed a slight rise in the WCA values after 30 days of ageing. The results obtained from XRD measurements in situ and after 30 days of ageing did not show the formation of an additional phase. The obtained morphology showed that the created dual surface roughness can effectively reduce bacterial adhesions and growth, as indicated by the performed antimicrobial tests.

There appears to be a distinct difference between the optical appearance after laser processing in the case of LIPSSs and micro-metre structures. In the case of LIPSSs, the material surface remains quite shiny without losing its mirror-polished properties. However, when micro-metre texturing is performed, a darkening of the processed surface is monitored without any shine. This finding leads to the conclusion that, by applying the correct laser parameters, the preservation of surface initial optical properties can be achieved to a greater extent while simultaneously affecting surface antimicrobial characteristics.

The current research demonstrated that, in order to achieve a more permanent decrease in surface contamination, the standardisation of the proposed experimental approach has to be obtained in order to gain reliable and qualitative results for surface antimicrobial activity.

## Figures and Tables

**Table 1:** Experimental conditions used for sample processing.

Group №	Scanning Velocity V (mm/s)	Laser Power (mW)
G1	0.1, 0.2, 0.5, 1, 3.44, 5.16, 7.6, 11.4, 32	20
G2	0.1, 0.2, 0.5, 1, 3.44, 5.16, 7.6, 11.4, 32	40

**Table 2:** Average area surface roughness (Sa) and line roughness (Ra) after the laser processing of stainless steel at two laser powers and scanning velocities ranging from V = 0.1 mm/s to 32 mm/s.

Irradiation Conditions	Ra—Averaged (Rpm) (µm)	Sa—Averaged (µm)	Distance b/n Heights Averaged (µm)	Distance b/n Rows Averaged (µm)	Depth of Grooves Averaged (µm)
Reference SS sample	-	0.04839	-	-	-
P = 20 mW, V = 0.1 mm/s	4.429	3.791	35.57	39.63	17.48
P = 20 mW, V = 0.2 mm/s	3.652	1.603	49.58	52.15	8.831
P = 20 mW, V = 0.5 mm/s	0.873	1.039	53.26	67.93	4.97
P = 20 mW, V = 1 mm/s	0.377	0.482	62.08	42.11	2.36
P = 20 mW, V = 3.44 mm/s	0.2459	0.1254	24.84	29.65	1.08
P = 20 mW, V = 5.16 mm/s	0.2592	0.1838	21.2	32.01	1.04
P = 20 mW, V = 7.6 mm/s	0.272	0.1717	20.43	37.69	0.98
P = 20 mW, V = 11.4 mm/s	0.2737	0.1925	16.49	40.25	0.81
P = 20 mW, V = 32 mm/s	0.2801	0.2412	13.92	42.66	0.69
P = 40 mW, V = 3.44 mm/s	0.2306	0.1983	17.53	37.95	1.2
P = 40 mW, V = 5.16 mm/s	0.2089	0.1334	16.4	39.18	1.01
P = 40 mW, V = 7.6 mm/s	0.2449	0.1594	14.8	40.12	0.97
P = 40 mW, V = 11.4 mm/s	0.2742	0.1755	11.01	42.02	0.89
P = 40 mW, V = 32 mm/s	0.2994	0.1945	9.96	43.97	0.74

## Data Availability

Not applicable.
